# Integration of a Multi-Camera Vision System and Strapdown Inertial Navigation System (SDINS) with a Modified Kalman Filter

**DOI:** 10.3390/s100605378

**Published:** 2010-05-28

**Authors:** Neda Parnian, Farid Golnaraghi

**Affiliations:** Mechatronic Systems Engineering, School of Engineering Science, Simon Fraser University, 250–13450 102nd Avenue, Surrey, BC V3T 0A3, Canada; E-Mail: neda_parnian@sfu.ca

**Keywords:** integration of vision system and SDINS, Extended Kalman Filter, Indirect Kalman Filter, strapdown inertial navigation system, tool positioning

## Abstract

This paper describes the development of a modified Kalman filter to integrate a multi-camera vision system and strapdown inertial navigation system (SDINS) for tracking a hand-held moving device for slow or nearly static applications over extended periods of time. In this algorithm, the magnitude of the changes in position and velocity are estimated and then added to the previous estimation of the position and velocity, respectively. The experimental results of the hybrid vision/SDINS design show that the position error of the tool tip in all directions is about one millimeter RMS. The proposed Kalman filter removes the effect of the gravitational force in the state-space model. As a result, the resulting error is eliminated and the resulting position is smoother and ripple-free.

## 1. Introduction

It is well known that inertial navigation sensors have drifts. There are two components in the inertial sensor drift: bias stability and bias variability. These components are involved in double integration in position calculation; so after a while, the output of the Inertial Navigation System (INS) is not reliable. Since these factors are involved in the inertial navigation computing task, they cause unavoidable drift in orientation and position estimation. Removing the drift of inertial navigation systems requires that the sensors be assisted with other resources or technologies such as Global Positioning Systems (GPS) [1,2], vision systems [3–5], or odometers [6,7].

The use of Kalman filters is a common method used in the data fusion technique. The Kalman filter is a powerful method for improving the output estimation and reducing the effect of sensor drift. However, sensor integration is based on Kalman filtering, but different types of Kalman filters are being developed in this area [8–14].

In the past, the three-dimensional attitude representations were applied, but these representations are singular or discontinuous for certain attitudes [15]. As a result, the quaternion parameterization was proposed, which has the lowest dimensional possibility for a globally non-singular attitude representation [16,17].

In aided inertial motion tracking applications, the state variables of a Kalman filter usually take one of two forms: first, the sensed engineering quantities, that is acceleration, velocity, and attitude, *etc.*; and second, the errors of these quantities. The first form is used by Centralized Kalman Filter [14], Unscented Kalman Filter [18–20], Adaptive Kalman Filter [10,21], and Sigma-point Extended Kalman Filter [22], while the second is used by Indirect Kalman Filter [23–25].

A Kalman filter that operates on the error states is called an indirect or a complementary Kalman filter. The optimal estimates of the errors are then subtracted from the sensed quantities to obtain the optimal estimates. Since the 1960s, the complementary Kalman filter has become the standard method of integrating non-inertial with inertial measurements in aviation and missile navigation. This method requires dynamic models for both the navigation variable states and the error states [26].

This research develops an EKF which offers the estimation of the changes in the state variables. Then the current estimated values of changes in the variables are added to the previous estimation values of the position and velocity, respectively. According to the general equations of the SDINS, the constant value of the gravitational force is removed from the resulted equations and the resulting error from the uncertainty value of the gravitational force is eliminated.

## 2. Kinematics of Strapdown Inertial Navigation Systems Using Quaternions

Inertial navigation systems are typically employed to provide the present position and heading of a moving vehicle with respect to the known and fixed reference frame. An inertial navigation system localizes the vehicle by measuring the linear and angular components of its motion using inertial sensors and with knowing the initial value of its position and attitude.

Since Microelectromechanical System (MEMS) techniques provide the opportunity to manufacture miniature inertial sensors inexpensively, this has led to the development of Strapdown Inertial Navigation Systems (SDINS) for new applications such as medicine [27,28], industry [29,30], robotics [31,32], and sports [33].

In SDINS, MEMS-based inertial sensors are mounted rigidly on the body of a moving object [34] to provide the applied forces to and the turning rates of the object, while accelerometers and angular rate gyros are parallel to the axis of the body. Because of the measuring of inertial components in the body frame, a set of equations must be derived to compute the position and attitude with respect to the known navigation reference frame.

**Figure 1.**
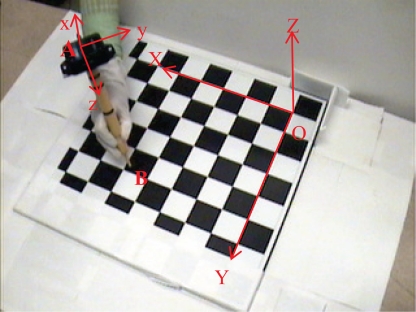
Hand-held tool and assigned reference frames.

Motion analysis of a moving rigid body provides a set of equations determining its trajectory, speed, and attitude. Since the Inertial Measurement Unit (IMU) measures the inertial components of the connected point, the acceleration and velocity of other points on the body can be computed relatively. As shown in Figure 1, the IMU is attached to the bottom of the tool when the position of the tool tip is desired.

As the tool is rotating and translating, the body reference frame shown in Figure 1 is relocating with respect to the fixed navigation frame. The relative acceleration of the point B [35] is computed as:

(1) $$a_{B}=a_{A}+\dot{\omega }\times r_{B/A}+\omega \times \left(\omega \times r_{B/A}\right)+2\omega \times {\left(v_{B/A}\right)}_{b}+{\left(a_{B/A}\right)}_{b}$$

where *a_A_* and *a_B_* represent acceleration of the points A and B; *r_B/A_*, (*v_B/A_*)*_b_*, and (*a_B/A_*)*_b_* denote the relative position, velocity, and acceleration of point B with respect to point A measured in the body frame; and ω̇ and ω are angular acceleration and angular velocity of the body frame.

Since both point A and point B are located on the tool and moving along the tool, the relative acceleration and velocity of point B with respect to A is zero, and Equation (1) is rewritten as:

(2) $$a_{B}=a_{A}+\dot{\omega }\times r_{B/A}+\omega \times \left(\omega \times r_{B/A}\right)$$

In order to transform the acceleration of the tool tip from the body frame into the North-East-Down (NED) frame [34], the cosine direction matrix must be computed from Equation (3):

(3) $${\dot{C}}_{b}^{n}=C_{b}^{n}\Omega_{\mathrm{nb}}^{n}$$

where $C_{b}^{n}$ and $\Omega_{\mathrm{nb}}^{n}$ denote the cosine direction matrix and the skew symmetric form of the body rate with respect to the navigation frame. The three-dimensional Euler angles representations were applied for attitude estimation in the SDINS, but these representations are singular or discontinuous for certain attitudes [15]. Since the quaternion parameterization has the lowest dimensional possibility for a globally non-singular attitude representation [16,17], the quaternion is generally used for attitude estimation in the SDINS.

The transformation matrix $C_{b}^{n}$ is related to the corresponding quaternion *q* = [*q*_1_ *q*_2_ *q*_3_ *q*_4_]*^T^*:

(4) $$C_{b}^{n}\;(q)=2\times \left[\begin{array}{ccc} q_{1}^{2}+q_{2}^{2}-0.5 & q_{2}q_{3}-q_{1}q_{4} & q_{2}q_{4}+q_{1}q_{3}\\ q_{2}q_{3}+q_{1}q_{4} & q_{1}^{2}+q_{3}^{2}-0.5 & q_{3}q_{4}-q_{1}q_{2}\\ q_{2}q_{4}-q_{1}q_{3} & q_{3}q_{4}+q_{1}q_{2} & q_{1}^{2}+q_{4}^{2}-0.5 \end{array}\right]$$

Therefore Equation (3) can be changed to Equation (5) as:

(5) $$\dot{q}=\frac{1}{2}q\Omega_{\mathrm{nb}}^{n}$$

Moreover, the gravity compensation is required since the accelerometers measure the local gravitational force. As a result, the acceleration of point B with respect to the navigation frame is calculated as:

(6) $${\left(a_{B}\right)}_{n}=C_{b}^{n}\left\{f_{A}+\dot{\omega }\times r_{B/A}+\omega \times \left(\omega \times r_{B/A}\right)\right\}+g_{n}$$

where *f_A_* represents the applied forces measured in the body frame by accelerometers, and *g_n_* denotes the gravity vector expressed in the navigation frame, [0 0 9.81]*^T^*.

As a result, the state space equations of the system can be finalized as:

(7) $$\begin{array}{l} {\dot{x}}^{n}=v^{n}\\ {\dot{v}}^{n}=C_{b}^{n}\;\{f_{A}+\dot{\omega }\times r_{B/A}+\omega \times (\omega \times r_{B/A})\}+g_{n}\\ \dot{q}=\frac{1}{2}\Lambda_{\mathrm{nb}}^{n}\;q=\frac{1}{2}Q(q)\;\left[\begin{array}{c} 0\\ \omega \end{array}\right]\\ \Lambda_{\mathrm{nb}}^{n}=\left[\begin{array}{ll} 0 & -\omega^{T}\\ \omega & -\Omega_{\mathrm{nb}}^{n} \end{array}\right] \end{array}$$

where *x^n^* and *v^n^* stand for the position and velocity of the tool tip with respect to the navigation frame, and *Q(q)* is the 4 × 4 real matrix representation of a quaternion vector.

The navigation frame and the body frame shown in Figure 1 are rotating with the Earth as well. According to relative motion equations, the acceleration of point A in the Earth frame is:

(8) $$a_{A}=a_{0}+{\dot{\omega }}_{E}\times r_{A/0}+\omega_{E}\times \left(\omega_{E}\times r_{A/0}\right)+2\omega \times {\left(v_{A/0}\right)}_{n}+{\left(a_{A/0}\right)}_{n}$$

where point O is the origin of the navigation frame. Since the navigation frame is fixed to the ground, then the relative acceleration of the navigation frame with respect to the Earth, *a_0_*, is zero. The angular velocity of the Earth is constant and nearly 7.3 × 10^−5^ rad/s [37], as a result the angular acceleration of the Earth is zero [36]. Since the relative position and velocity of point A with respect to point O is too small because of description of on-hand application, the effect of Coriolis and the centripetal acceleration terms in Equation (8) is too small to be detected by available accelerometers; therefore:

(9) $$a_{A}={\left(a_{A/0}\right)}_{n}$$

which means the acceleration of point A with respect to the Earth reference frame is its acceleration with respect to the navigation frame.

## 3. Vision System

**Figure 2.**
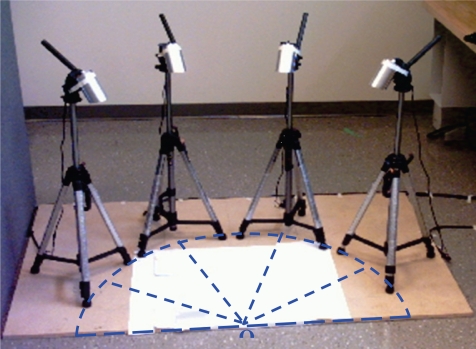
Experimental setup for the multi-camera vision system.

In this research, a vision system is proposed which includes four CCD cameras located on an arc to expand the domain of the field of view, see Figure 2. In order to find the Cartesian mapping grid for transforming 2D positions in the cameras’ image plane to the corresponding 3D position in the navigation frame, the single camera calibration for each camera and the stereo camera calibration for each two adjacent cameras are required.

**Figure 3.**
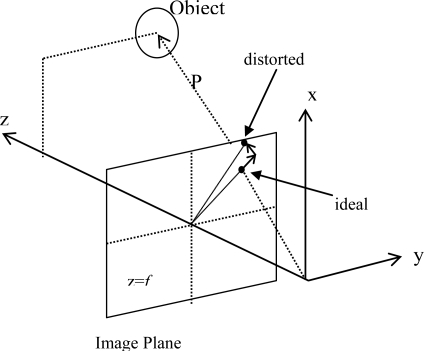
Camera imaging model.

The calibration of the vision system provides the intrinsic and extrinsic parameters of the cameras [38] in order to map a 2D point on the image planes to the 3D point in the world coordinate system. The estimation of camera parameters requires a single camera imaging model, as shown in Figure 3.

The camera lens distortion causes two radial and tangential displacements [39]. The longer distance from the center of the image plane initiates the larger displacement, when the distance of a point $p={\left[\begin{array}{cc} x & y \end{array}\right]}^{T}={\left[\begin{array}{cc} \frac{P_{x}}{P_{z}} & \frac{P_{y}}{P_{z}} \end{array}\right]}^{T}$ on the image plane is defined as *r*^2^ = (*x*)^2^ + (*y*)^2^.

Considering two vectors α and β as the radial and tangential distortion factors of a camera, the distortions can be calculated as [40]:

(10) $$\begin{array}{l} d_{r}=\left(1+\alpha_{x}\;r^{2}+\alpha_{y}\;r^{4}\right)\\ d_{t}=\left[\begin{array}{c} 2\beta_{x}\;\mathrm{xy}+\beta_{y}\;(r^{2}+2x^{2})\\ \beta_{x}\;(r^{2}+2x^{2})+2\beta_{y}\;\mathrm{xy} \end{array}\right] \end{array}$$

Consequently, the projection of each point in the world coordinate system into the image plane is:

(11) $$p=\left[\begin{array}{c} f_{1}d_{r}\;x\\ f_{2}d_{r}\;y \end{array}\right]+\left[\begin{array}{c} d_{t,x}\\ d_{t,y} \end{array}\right]+\left[\begin{array}{c} N_{x}\\ N_{y} \end{array}\right]$$

where the vector *N* is a zero-mean Gaussian random measurement noise, and *f*_1_ and *f*_2_ denote the focal length factors of the lens. In fact, *f*_1_ and *f*_2_ are related to the focal length and the dimension of the pixels:

(12) $$\begin{array}{l} f_{1}=\frac{f.\;s_{u}}{d_{\mathrm{px}}}\\ f_{2}=\frac{f}{d_{\mathrm{py}}} \end{array}$$

where *f* is the focal length; *d_px_* and *d_py_* refer to center-to-center distance between adjacent sensor elements in x and y directions, respectively; and *s_u_* represents the image scale factor [41], therefore:

(13) $$p=f\;d_{r}\;\left[\begin{array}{c} {\mathrm{ks}}_{u}\frac{x}{z}\\ l\frac{y}{z} \end{array}\right]+\left[\begin{array}{c} d_{t,x}\\ d_{t,y} \end{array}\right]+\left[\begin{array}{c} N_{x}\\ N_{y} \end{array}\right]$$

where $\frac{1}{k}$ and $\frac{1}{l}$ denote the dimension of a pixel on the image plane.

According to the camera model obtained in Equation (13), the geometric parameters *f*, *s_u_*, α, and β can be estimated by capturing enough images while the coordinate of each 3D point *P* and its 2D projected point *p* are known in calibration grids:

(14) $$p=\frac{1}{z}\left[\begin{array}{ccc} f\;d_{r}\;{\mathrm{ks}}_{u} & 0 & 0\\ 0 & f\;d_{r}\;l & 0\\ 0 & 0 & 1 \end{array}\right]\;\left[\begin{array}{c} x\\ y\\ 1 \end{array}\right]+\left[\begin{array}{c} N_{x}\\ N_{y} \end{array}\right]=\frac{1}{z}\mathrm{MP}+N$$

Applying the parameter estimation method [34,42] to Equation (11) gives the geometric parameters of a camera. Furthermore, the transformation matrix for each two adjacent cameras is computed by substituting the equations of the coordinate system transformation into Equation (11) for each corresponding projected point.

In order to localize the tool tip, the edge detection and boundary extraction must be applied to every single frame from each camera. Obtaining the edge of the tool tip requires applying a thresholding technique. Each pixel is detected as an edge if its gradient is greater than the threshold. In this paper, the threshold is chosen as the boundary pixels of the tool tip are detected as the edge positions. Since the size of the tool tip is about a few pixels, then an adaptive thresholding technique is applied to remove the noise pixels around the tool tip as much as possible. For this purpose, a masking window is chosen around the initial guess of the position of the tool tip. Then, a fixed threshold is chosen which select pixels that their value is above the 80% of the value of all pixels of the image. If the boundary detection technique can identify the boundary of the tool tip, then it shows that the threshold selection is appropriate. Otherwise, the previous threshold is reduced by 5%, and this procedure is run recursively to find the proper threshold. Afterwards, the opening morphologic operation followed by closing operation is applied to simplify and smooth the shape of the tool tip. Finally, the boundary of the tool tip can be detected and extracted by using the eight-connected neighbors’ technique.

## 4. Modified Kalman Filter

**Figure 4.**
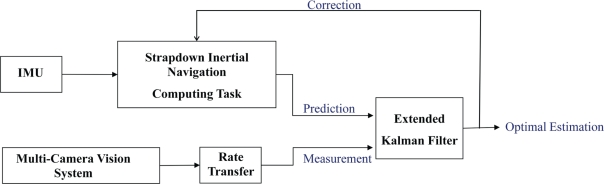
Integration of SDINS and vision system using EKF.

The integrated navigation technique employs two or more independent sources of navigation information with complementary characteristics to achieve an accurate, reliable, and low-cost navigation system. Figure 4 shows a block diagram of the integration of the multi-camera vision system and the inertial navigation system:

Typically, Extended Kalman Filter (EKF) is applied by combining two independent estimates of a nonlinear variable [43]. The continuous form of a nonlinear system is described as:

(15) $$\begin{array}{l} \dot{x}(t)=f(x(t),\;t)+G(t)\eta (t)\\ z(t)=h(x(t),\;k)+n \end{array}$$

Since the measurements are practically provided at discrete intervals of time, it is appropriate to express the system modeling in the form of discrete differential equations:

(16) $$\begin{array}{l} x_{k+1}=\phi_{k}\;x_{k}+\eta_{k}\\ z_{k+1}=H_{k+1}\;x_{k+1}+n_{k+1} \end{array}$$

where:

(17) $$\begin{array}{l} \phi_{k}=\text{exp}\left[F(t_{k+1}-t_{k})\right]\\ F(t_{k})\equiv {\frac{\partial f}{\partial x}|}_{x={\overset{︷}{x}}_{k}}\;\;\;\mathrm{and}\;H_{k}\equiv {\frac{\partial f}{\partial x}|}_{x={\overset{︷}{x}}_{k}} \end{array}$$

Therefore the two set of equations involving the prediction and updating of the state of the system are defined as:

(18) $$\begin{array}{ccc} {\tilde{x}}_{k+1} & = & f\left({\overset{︷}{x}}_{k},\;k\right)\\ \tilde{P} & = & F_{k}\;P_{k}\;F_{k}^{T}+G_{k}\;R_{k}\;G_{k}^{T}\\ {\overset{︷}{x}}_{k+1} & = & x_{k+1}+K\left[z_{k+1}-h\;({\tilde{x}}_{k},\;k)\right]\\ P_{k+1} & = & \tilde{P}-{\mathrm{KH}}_{k}\;\tilde{P}\\ K & = & \tilde{P}H_{k}^{T}\;{\left(S_{k}+H_{k}\;\tilde{P}H_{k}^{T}\right)}^{-1} \end{array}$$

where the system noise and the measurement noise are zero mean with known covariance *R* and *S*, respectively.

According to Equations (7), (17), and (18), the discrete form of the system is developed as:

(19) $$\begin{array}{ccc} x_{k+1} & = & x_{k}+T_{i}\;v_{k}\\ v_{k+1} & = & v_{k}+T_{i}\;(C_{k}\;a_{k}+g_{n})\\ q_{k+1} & = & (I+0.5T_{i}\Omega )q_{k}\\ \;\;\;a & = & f_{A}+\dot{\omega }\times r_{B/A}+\omega \times (\omega \times r_{B/A}) \end{array}$$

where *T_i_* is the sampling rate of the inertial sensors. In this research, instead of estimating the actual value of these quantities, we propose to estimate how much the position and the velocity will be changed; that is:

(20) $$\begin{array}{l} \Delta x_{k+1}=x_{k+1}-x_{k}=\Delta x_{k}+T_{i}\Delta v_{k}\\ \Delta v_{k+1}=v_{k+1}-v_{k}=\Delta v_{k}+T_{i}\;(\Delta C_{k}\;a_{k-1}+C_{k}\;\Delta a_{k}) \end{array}$$

As a consequence, the computation of the velocity is independent of the gravitational force in the new state-space model. In fact, the error caused by inaccurate value of the gravitational force in the new state-space model is completely eliminated.

The inertial sensor noise is theoretically modeled with a zero-mean Gaussian random process. In practice, the average of the noise is not absolutely zero. Due to the inherent characteristic of the Gaussian random process, the discrete difference of a zero-mean Gaussian random process is also a zero-mean Gaussian random process with very lower actual mean while its variance is twice of the variance of the original process. As a result, the drift resulting from the input noise is reduced and a smooth positioning is expected.

The equation of the INS with the state vector *X* = [Δ*x* Δ*v q*]*^T^* can be reformulated as:

(21) $$\left[\begin{array}{c} \Delta^{\cdot }x\\ \Delta^{\cdot }v\\ \dot{q} \end{array}\right]=\left[\begin{array}{ccc} 0 & I & 0\\ 0 & 0 & 0\\ 0 & 0 & 0.5\Lambda \end{array}\right]\;\left[\begin{array}{c} \Delta x\\ \Delta v\\ q \end{array}\right]+\left[\begin{array}{c} 0\\ \Delta \mathrm{Ca}+C\Delta a\\ 0 \end{array}\right]+\left[\begin{array}{cc} 0 & 0\\ 0 & \Delta C+2C\\ 0.5Q(q) & 0 \end{array}\right]\eta (t)$$

or:

(22) $$\left[\begin{array}{c} \Delta^{\cdot }r\\ \Delta^{\cdot }v\\ \dot{q} \end{array}\right]=\left[\begin{array}{c} \Delta v\\ \Delta \mathrm{Ca}+C\Delta a\\ 0.5\Lambda \;q \end{array}\right]+\left[\begin{array}{cc} 0 & 0\\ 0 & \Delta C+2C\\ 0.5Q(q) & 0 \end{array}\right]\;\eta (t)$$

Subsequently, the transition matrix [44] can be calculated as:

(23) $$F\equiv {\frac{\partial f}{\partial X}|}_{X=\overset{︷}{X}}=\left[\begin{array}{ccc} 0 & I & 0\\ 0 & 0 & \frac{\partial }{\partial q}(\Delta \mathrm{Ca}+C\Delta a)\\ 0 & 0 & 0.5\Lambda \end{array}\right]$$

By considering Δ*a* = [Δ_1_ Δ_2_ Δ_3_]*^T^*:

(24) $$\begin{array}{l} \frac{\partial }{\partial q}C\Delta a=2[\begin{array}{cc} q_{1}\Delta_{1}-q_{4}\Delta_{2}+q_{3}\Delta_{3} & q_{2}\Delta_{1}+q_{3}\Delta_{2}+q_{4}\Delta_{3}\\ q_{4}\Delta_{1}+q_{1}\Delta_{2}-q_{4}\Delta_{3} & q_{3}\Delta_{1}-q_{2}\Delta_{2}-q_{1}\Delta_{3}\\ -q_{3}\Delta_{1}+q_{2}\Delta_{2}+q_{1}\Delta_{3} & q_{4}\Delta_{1}+q_{1}\Delta_{2}-q_{2}\Delta_{3} \end{array}\\ \;\;\;\;\;\;\;\;\;\;\;\;\;\begin{array}{cc} -q_{3}\Delta_{1}+q_{2}\Delta_{2}+q_{1}\Delta_{3} & -q_{4}\Delta_{1}-q_{1}\Delta_{2}+q_{2}\Delta_{3}\\ q_{2}\Delta_{1}+q_{3}\Delta_{2}+q_{4}\Delta_{3} & q_{1}\Delta_{1}-q_{4}\Delta_{2}-q_{1}\Delta_{3}\\ -q_{1}\Delta_{1}+q_{4}\Delta_{2}-q_{3}\Delta_{3} & q_{2}\Delta_{1}+q_{3}\Delta_{2}+q_{4}\Delta_{3} \end{array}] \end{array}$$

Substituting $\dot{C}=\underset{\Delta t\to 0}{\text{lim}}\left(\frac{\Delta C}{\Delta t}\right)$, where Δ*t* = *T*, into Equation (3) leads to the following Equation:

(25) $$\Delta C=-T_{i}C\Omega$$

Therefore:

(26) $$\begin{array}{l} \frac{\partial }{\partial q}\;\Delta \mathrm{Ca}=\frac{\partial }{\partial q}(-T_{i}C\Omega a)=-T_{i}\frac{\partial }{\partial q}C\alpha \\ \alpha =\Omega a=\left[\begin{array}{c} \alpha_{1}\\ \alpha_{2}\\ \alpha_{3} \end{array}\right]=\left[\begin{array}{c} -\omega_{3}a_{2}+\omega_{2}a_{3}\\ \omega_{3}a_{1}-\omega_{1}a_{2}\\ \omega_{2}a_{1}+\omega_{1}a_{2} \end{array}\right] \end{array}$$

As a result of Equation (106):

(27) $$\begin{array}{l} \frac{\partial }{\partial q}C\alpha =2[\begin{array}{cc} q_{1}\alpha_{1}-q_{4}\alpha_{2}+q_{3}\alpha_{3} & q_{2}\alpha_{1}+q_{3}\alpha_{2}+q_{4}\alpha_{3}\\ q_{4}\alpha_{1}+q_{1}\alpha_{2}-q_{4}\alpha_{3} & q_{3}\alpha_{1}-q_{2}\alpha_{2}-q_{1}\alpha_{3}\\ -q_{3}\alpha_{1}+q_{2}\alpha_{2}+q_{1}\alpha_{3} & q_{4}\alpha_{1}+q_{1}\alpha_{2}-q_{2}\alpha_{3} \end{array}\\ \;\;\;\;\;\;\;\;\;\;\;\begin{array}{cc} -q_{3}\alpha_{1}+q_{2}\alpha_{2}+q_{1}\alpha_{3} & -q_{4}\alpha_{1}-q_{1}\alpha_{2}+q_{2}\alpha_{3}\\ q_{2}\alpha_{1}+q_{3}\alpha_{2}+q_{4}\alpha_{3} & q_{1}\alpha_{1}-q_{4}\alpha_{2}-q_{1}\alpha_{3}\\ -q_{1}\alpha_{1}+q_{4}\alpha_{2}-q_{3}\alpha_{3} & q_{2}\alpha_{1}+q_{3}\alpha_{2}+q_{4}\alpha_{3} \end{array}] \end{array}$$

Because the vision system as the measurement system provides the position of the tool tip, velocity can be computed by knowing the present and the previous position at each time step:

(28) $$\begin{array}{l} \tilde{z}=z+n=\left[\begin{array}{c} \tilde{x}\\ \tilde{v} \end{array}\right]\\ {\tilde{v}}_{l+1}=\frac{{\tilde{x}}_{l+1}-{\tilde{x}}_{l}}{T_{v}} \end{array}$$

where *T_v_* is the sampling rate of the cameras. Accordingly, the observation matrix would be:

(29) $$\begin{array}{c} \Delta z_{k+1}=\left[\begin{array}{ccc} I & 0 & 0\\ 0 & I & 0\\ 0 & 0 & 0 \end{array}\right]\;\left[\begin{array}{c} \Delta r_{k+1}\\ \Delta v_{k+1}\\ q_{k+1} \end{array}\right]+n_{k+1}\\ \Delta v_{k+1}=\frac{T_{i}}{2T_{v}}(\Delta r_{k+1}-\Delta r_{k}) \end{array}$$

## 5. Experimental Results

This section presents the experimental hardware setup and the result of applying the proposed EKF. The experimental hardware includes a 3DX-GX1 IMU from Microstrain, an IDS Falcon Quattro PCIe frame grabber from IDS Imaging Development Systems, and four surveillance IR-CCD cameras. The IMU contains three rate gyros and three accelerometers with a sampling rate of 100 Hz and with a noise density of 3.5 °/ $\sqrt{\mathrm{hour}}$ and 0.4 *mg*/*rms* $\sqrt{\mathrm{Hz}}$, respectively [45].

All cameras are connected through the frame grabber to a PC, which includes four parallel video channels able to capture images from four cameras simultaneously with a sampling rate of 20 fps. Since the multi-camera vision system is used as a measurement system, the camera calibration procedure must be performed primary. The intrinsic and extrinsic parameters of each camera are listed in Table 1.

**Figure 5.**
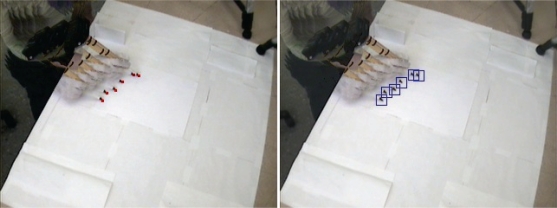
Tool tip tracking by Camera #1.

Once the calibration is completed, the vision system is ready to track the tool and measure the position of the tool tip by applying image processing techniques. Figure 5 demonstrates the result of the video tracking by one of the cameras.

It should be mentioned that a predesigned path is printed on the 2D plane and it is tried to be traced by the tool tip during its movement on the plane in order to compare the performance of proposed EKF and with the performance of the conventional EKF reported in [5].

The sensor fusion techniques allow us estimating the states variables of the system at the sampling rate of the sensor with the highest measurement rate. In this experiment, the sampling rate of cameras and inertial sensors are 20 fps and 100 Hz. As a result of sensor fusion, the measurement rate of the proposed integrated system is 100 Hz.

The classical EKF is applied in both switch and continues modes. In the switch mode, the estimation of the states variables is corrected whenever the measurement of the vision system is available. Otherwise, the states are estimated only based on the SDINS. In order to reduce the computational complexity of image processing algorithms, sensor fusion allows that the sampling rate of the vision system can be reduced to 10 fps and 5 fps. As illustrated in Table 2, the positioning error is increased by reducing the sampling rate of the cameras. In addition, the error in proposed EKF grows faster than the other methods; since this technique assumes that the rate of the changes in state variables is constant from one frame to another frame. So, this assumption cannot be valid in lower measurement rates.

Although, it is shown in Table 2 that the position error of the continuous EKF is less than the others; it should be mentioned that the position obtained by the multi-camera vision system still has errors compared with the predesigned path.

**Figure 6.**
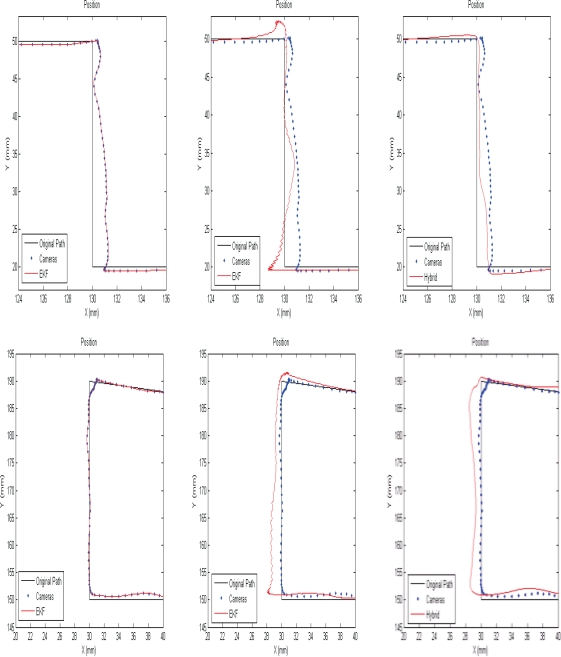
Estimated position by applying different estimation method: continuous EKF (left), Switch EKF (center), and proposed EKF (right); when the sampling rate of the cameras is 16 fps.

**Figure 7.**
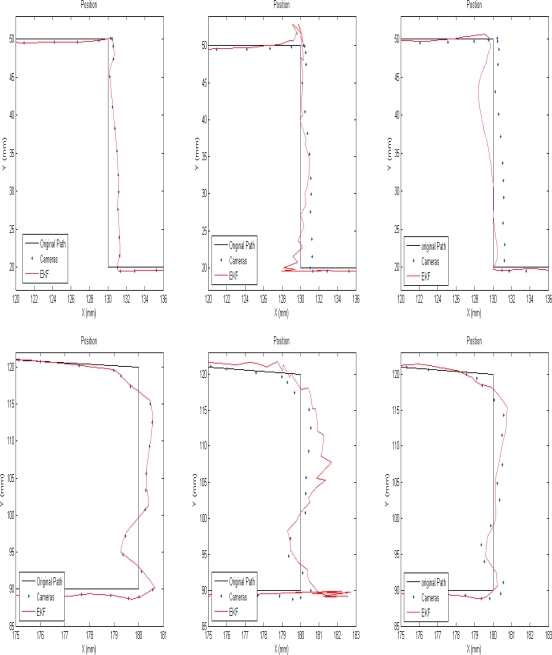
Estimated position by applying different estimation method: continuous EKF (left), Switch EKF (center), and proposed EKF (right); when the sampling rate of the cameras is 5 fps.

Figure 6 and Figure 7 compare the position resulting from each method at two different parts of the trajectory of the tool tip at two sampling rate of 16 fps and 5 fps. As shown, the camera path is traced smoothly by applying continuous EKF. Since the position is estimated in real-time, it is not possible to fit a curve between each two camera measurement without sensor fusion techniques.

The position resulting from switch EKF is crinkly due to the drift position in the SDINS and the wrinkles are amplified by decreasing the measurement rate of the cameras. The position estimated by the proposed EKF is smooth and ripple-free and this method tries to reduce the errors of the entire system compared with the predesigned path. As a result, the proposed EKF is suitable for the higher measurement rate; while the continuous EKF is recommended for the lower sampling rate. However, the error of inertial sensors resulting from noise and the common motion-dependent errors are compensated, but the remaining errors cause the position error estimation in the integrated system. In addition, the video tracking errors lead to the position estimation error as well.

## 6. Conclusions

This paper describes the use of the EKF to develop integration of the multi-camera vision system and inertial sensors. The sensor fusion techniques allow estimation of the state variables at the sampling rate of the sensor with the highest measurement rate. This helps to reduce the sampling rate of the sensors with high computational load.

The classical EKF is designed for nonlinear dynamic systems such as the strapdown inertial navigation system. The performance of the classical EKF is reduced by lowering the sampling rate of the cameras. When the sampling rate of the cameras is reduced, the rate of updating decreases and the system must rely more on the inertial sensors output for estimating the position. Because of the drift in the SDINS, the position error increases.

The modified EKF is proposed to obtain position estimation with less error. Furthermore, it removes the effect of the gravitational force in the state-space model. In fact, the error resulting from inaccuracy in the evaluation of the gravitational force is eliminated in the state-space model. In addition, the estimated position is smooth and ripple-free. However; the proposed EKF is not convincing at the lower measurement rate. The error of the estimated position results from inertial sensor errors, uncompensated common motion-dependent errors, attitude errors, video tracking errors, and unsynchronized data.

## Figures and Tables

**Table 1.** Intrinsic and extrinsic parameters.

	**Camera #1**	**Camera #2**	**Camera #3**	**Camera #4**
**Focal Length**	X: 400.69 pixels Y: 402.55 pixels	X: 398.51 pixels Y: 400.44 pixels	X: 402.00 pixels Y: 405.10 pixels	X: 398.74 pixels Y: 400.60 pixels
**Principal Point**	X: 131.12 pixels Y: 130.10 pixels	X: 152.74 pixels Y: 122.79 pixels	X: 144.77 pixels Y: 118.23 pixels	X: 136.90 pixels Y: 145.34 pixels
**Distortion Coefficients**	*K_r,x_*: −0.3494 *K_r,y_*: 0.1511 *K_t,x_*: 0.0032 *K_t,y_*: −0.0030	*K_r,x_*: −0.3522 *K_r,y_*: 0.1608 *K_t,x_*: 0.0047 *K_t,y_*: −0.0005	*K_r,x_*: −0.3567 *K_r,y_*: 0.0998 *K_t,x_*: −0.0024 *K_t,y_*: 0.0016	*K_r,x_*: −0.3522 *K_r,y_*: 0.0885 *K_t,x_*: 0.0024 *K_t,y_*: −0.0002
**Rotation Vector** **Wrt Inertial** **Reference Frame**	1.552265 2.255665 −0.635153	0.4686021 2.889162 −0.7405382	0.6128003 −2.859007 0.7741390	1.537200 −2.314144 0.4821106
**Translation Vector** **wrt Inertial** **Reference Frame**	729.4870 mm 293.6999 mm 873.3399 mm	385.2578 mm 625.1560 mm 840.7220 mm	−61.1933 mm 623.1377 mm 851.9321 mm	−365.5847 mm 289.6135 mm 848.5442 mm

**Table 2.** Positions estimated by different estimation methods are compared with the position estimated by the multi-camera vision system.

	**Proposed EKF**		**EKF (Switch)**		**EKF (Continuous)**	
**Cameras Measurement Rate**	**Error (RMS)**	**Variance**	**Error (RMS)**	**Variance**	**Error (RMS)**	**Variance**
16 fps	0.9854	0.1779	1.0076	0.7851	0.4320	0.1386
10 fps	1.0883	0.3197	1.2147	0.8343	0.5658	0.2149
5 fps	1.4730	1.5173	1.3278	0.8755	0.7257	0.8025
